# Student Characteristics and ICT Usage as Predictors of Computational Thinking: An Explainable AI Approach

**DOI:** 10.3390/jintelligence13110145

**Published:** 2025-11-11

**Authors:** Tongtong Guan, Liqiang Zhang, Xingshu Ji, Yuze He, Yonghe Zheng

**Affiliations:** 1 Research Institute of Science Education, Beijing Normal University, Beijing 100875, China; 202431010058@mail.bnu.edu.cn (T.G.); 202421010165@mail.bnu.edu.cn (X.J.); 2 Faculty of Psychology, Beijing Normal University, Beijing 100875, China; zlq2023@mail.bnu.edu.cn

**Keywords:** computational thinking, ICT, explainable AI, educational data mining

## Abstract

Computational thinking (CT) is recognized as a core competency for the 21st century, and its development is shaped by multiple factors, including students’ individual characteristics and their use of information and communication technology (ICT). Drawing on large-scale international data from the 2023 cycle of the International Computer and Information Literacy Study (ICILS), this study analyzes a sample of 81,871 Grade 8 students from 23 countries and one regional education system who completed the CT assessment. This study is the first to apply a predictive modeling framework that integrates two machine learning techniques to systematically identify and explain the key variables that predict CT and their nonlinear effects. The results reveal that various student-level predictors—such as educational expectations and the number of books at home—as well as ICT usage across different contexts, demonstrate significant nonlinear patterns in the model, including U-shaped, inverted U-shaped, and monotonic trends. Compared with traditional linear models, the SHapley Additive exPlanations (SHAP)-based approach facilitates the interpretation of the complex nonlinear effects that shape CT development. Methodologically, this study expands the integration of educational data mining and explainable artificial intelligence (XAI). Practically, it provides actionable insights for ICT-integrated instructional design and targeted educational interventions. Future research can incorporate longitudinal data to explore the developmental trajectories and causal mechanisms of students’ CT over time.

## 1. Introduction

Computational thinking (CT) has emerged as a critical domain of inquiry that has attracted substantial attention in academic communities worldwide. CT is now recognized as a central theme in global educational reform and technological innovation (Wing 2006). Rather than being confined to computer science, CT represents a general problem-solving approach applicable across a wide range of disciplines, including science, engineering, mathematics, and the social sciences (Shute et al. 2017). In the era of artificial intelligence (AI) and big data, the development of students’ CT is crucial for their ability to navigate and succeed in a future society (Shute et al. 2017). In response to this need, numerous countries have integrated CT into their K–12 education systems as a strategy to enhance students’ digital literacy and technological competitiveness (Fraillon and Rožman 2025). Educational policies worldwide increasingly regard CT as a core competence of basic education aimed at preparing students for a rapidly digitalized and intelligent society (Yadav et al. 2016), which, in this study, is conceptualized as a technologically advanced society characterized by digitalization, data-driven innovation, and AI integration in education (Cukurova 2025; Zawacki-Richter et al. 2019). Despite these initiatives, substantial individual differences persist in students’ CT proficiency, which reflects the complex interplay of multiple influencing factors. These differences are shaped not only by intrinsic learner characteristics but also by external variables such as educational environments, socioeconomic status, and patterns of information and communication technology (ICT) use (Fraillon and Rožman 2025). First, individual cognitive ability and learning preferences may play a role in the development of CT (Fraillon and Rožman 2025). Second, the educational context and instructional methods significantly influence CT development. Access to high-quality teaching resources, a supportive learning atmosphere, and effective pedagogical strategies can all foster growth in CT. For example, socioeconomic status may indirectly shape students’ CT development by influencing their access to digital technologies, learning support, and an enriched cognitive environment (Fraillon et al. 2020; Tan and Hew 2019). Furthermore, social and technological contexts exert considerable influence. The widespread adoption of computer technology and the promotion of coding education have been shown to contribute positively to students’ CT development (Grover and Pea 2018). Importantly, several dimensions of CT can also be cultivated without the use of digital technology. For instance, the CS Unplugged initiative provides widely adopted unplugged activities that foster problem-solving and algorithmic thinking skills in non-digital contexts. Thus, understanding individual differences in CT and uncovering the multilevel mechanisms behind its development holds both theoretical significance and practical value for promoting educational equity and precision teaching.

Despite the increasing recognition of CT as a strategic competence in K–12 education, research on its influencing factors remains limited in several ways. Most empirical studies rely on traditional statistical models, such as multiple regression or structural equation modeling (SEM), which assume linear and independent effects. These assumptions often fail to capture the complex, nonlinear interactions among the cognitive, behavioral, and environmental variables that shape CT development (Román-González et al. 2017), and they potentially overlook key predictors. In addition, although some studies have examined the relationship between CT and ICT usage, much of this evidence is based on small-scale or qualitative research. Prior work has tended to emphasize instructional interventions, with insufficient attention given to how student-level factors—such as cognitive ability, digital literacy, and access to learning resources—affect CT through developmental pathways. Addressing these limitations requires methods that can model both direct and interaction effects in high-dimensional datasets.

To fill this gap, this study integrates large-scale International Computer and Information Literacy Study (ICILS 2023) data with explainable AI (XAI) methods. This approach reflects the recent shifts in CT research that highlight not only cognitive factors but also broader contextual factors, including curriculum structures and digital equity. Prior work has explored individual predictors, but few studies have systematically examined how student characteristics interact to predict CT performance. Moreover, linear modeling approaches are limited in capturing such dynamics. Therefore, we employ a machine learning framework designed to detect nonlinear interactions while maintaining interpretability. Specifically, we draw on XAI, with SHapley Additive exPlanations (SHAP) providing model-agnostic insights into variable importance and interaction effects (Lundberg and Lee 2017). This approach enables a more nuanced understanding of how diverse student factors shape CT and supports the design of targeted interventions.

Grounded in the ICILS 2023 framework of computational thinking, the present study formulates the following research questions:

**RQ1.**  *Within the ICILS 2023 framework, which student background characteristics and ICT usage variables are most strongly associated with computational thinking?*

**RQ2.**  *How do these most influential factors shape or explain students’ computational thinking performance?*

## 2. Literature Review

### 2.1. Computational Thinking

The concept of CT was first introduced by Wing (2006), who emphasized that CT is not merely a programming skill but also a cross-disciplinary cognitive approach that enables individuals to solve complex problems efficiently. CT encompasses a suite of cognitive skills, such as logical reasoning, pattern recognition, data analysis, and algorithm design, which allow individuals to effectively model and solve problems using computational tools (Wing 2006). Furthermore, CT emphasizes scalability, which refers to its applicability across problems of varying complexity. Some scholars have further argued that CT possesses a high degree of scalability that enables individuals to flexibly address problems of differing complexity through algorithmic processes and mathematical modeling, thereby facilitating system optimization and predictive reasoning (Schwartz et al. 2024). This perspective extends the applicability of CT in educational practice and underscores its value as a domain-general problem-solving tool (Sudadi et al. 2023). In recent years, the study of CT has evolved beyond technical skills to encompass more complex cognitive and practical competencies (Rafiq et al. 2023). Shute et al. proposed that CT involves not only operational proficiency with computational tools but also data-driven reasoning, metacognitive regulation, and the ability to transfer and integrate CT strategies across disciplines (Shute et al. 2017). Although Shute et al. highlighted the lack of consistent assessments as a challenge in CT research, this study addresses this concern by adopting the empirically validated definition and measurement framework developed in ICILS 2023. This framework defines CT as a domain-specific problem-solving competence in digital contexts that comprises two dimensions: conceptualizing problems and operationalizing solutions (Fraillon and Rožman 2025).

The International Association for the Evaluation of Educational Achievement (IEA), through its 2023 cycle of ICILS, proposed a comprehensive CT assessment framework that serves as an authoritative reference for global K–12 education. ICILS 2023 offers a systematic definition of CT and incorporates individual cognitive dimensions and contextual factors such as curriculum design, instructional practices (e.g., interdisciplinary integration), and ICT usage (e.g., frequency of coding activities). These considerations reveal the multifactorial influences that shape CT development. Within the ICILS framework, CT is positioned as a core competency essential for solving complex problems in a digital society. Specifically, it is defined as the ability to use fundamental concepts from computer science to reason about and solve problems encountered in real-world contexts, including the design of systems and the understanding of human behavior. Emphasis is placed on the cognitive processes involved in identifying computable aspects of real-world problems and developing executable algorithmic solutions (Fraillon and Rožman 2025). The ICILS framework conceptualizes CT as a domain-general problem-solving strategy that involves key dimensions such as problem decomposition, abstract modeling, and algorithm design, evaluation, and optimization. These are further delineated into the five interrelated cognitive processes of abstraction, algorithmic thinking, automation, decomposition, and debugging. The framework not only highlights the measurement of cognitive abilities but also integrates systemic educational variables—such as curriculum content and pedagogical practices—and individual student characteristics—such as socioeconomic background and ICT proficiency—into a multi-layered model of influencing factors. Notably, the ICILS 2023 assessment adopts a task-based and context-simulated approach that uses realistic problem-solving scenarios to evaluate students’ CT in non-programming contexts. This design addresses the limitations in traditional programming-focused assessments by capturing students’ ability to transfer cognitive skills and apply CT in interdisciplinary, authentic settings.

Accordingly, CT has become a key indicator of students’ 21st-century competencies. Its conceptualization has shifted from skill-based to thought-based and from technical utility to a broader problem-solving strategy. The ICILS 2023 framework provides a theoretically grounded and empirically supported basis for the structured assessment and educational intervention of CT while highlighting the critical roles of individual background and ICT usage in the development of CT.

### 2.2. Research Progress on the Factors That Influence CT

As a core competence of 21st-century education, CT is shaped by the complex interplay of multiple factors (Rao and Bhagat 2024; Sun et al. 2024). Recent research on K–12 education has revealed the multidimensional mechanisms underlying CT development, highlighting the roles of students’ cognitive abilities, socioeconomic backgrounds, educational environments, and exposure to technology (Stewart et al. 2021; Sudadi et al. 2023). At the cognitive level, logical reasoning, mathematical ability, and metacognitive regulation are widely regarded as foundational to CT (Agbo et al. 2023). Current research trends suggest a gradual shift in focus from curriculum design and instructional tools toward learner characteristics (Hsu et al. 2018; Nouri et al. 2020). Among these factors, students’ technological foundation—particularly their ICT usage competencies—has been identified as a key predictor of CT performance. Empirical studies have shown that students with higher digital literacy and stronger internet self-efficacy tend to perform better on CT assessments (Román-González et al. 2017). Moreover, students with basic programming experience and practical computer skills often exhibit significant advantages in problem decomposition, algorithm design, and debugging (Grover and Basu 2017; Weintrop et al. 2016). Although the relationship between internet usage frequency and CT outcomes remains inconclusive, there is growing evidence that the quality of ICT usage is a more robust predictor of CT development than its quantity (De Medio et al. 2020; Durak and Saritepeci 2018). For example, students’ ability to integrate ICT effectively into academic activities—such as information retrieval, data analysis, or project-based learning—has been shown to significantly enhance CT performance (Voogt et al. 2015). At the personal background level, factors such as gender, grade level, academic achievement, and the home technology environment also contribute meaningfully to CT outcomes (Hu 2024). In addition, older students and those with stronger science, technology, engineering, and mathematical (STEM) backgrounds generally demonstrate higher CT, indicating a cumulative developmental trajectory (Durak and Saritepeci 2018; Özgür 2020). Socioeconomic status indirectly influences CT by shaping students’ opportunities to access and engage with ICT; those from under-resourced backgrounds often face a “disadvantaged starting point” in CT development (Ezeamuzie 2024; Ezeamuzie et al. 2024).

The ICILS studies of 2018 and 2023 further expanded the conceptualization of contextual influences on CT by incorporating systemic factors such as curriculum structure, instructional practices, and ICT usage contexts into their background frameworks (Fraillon et al. 2019). This holistic approach emphasizes the need to go beyond individual cognition and consider broader educational and technological ecosystems when analyzing CT development. Nevertheless, despite a growing body of research that identifies the relevant predictors of CT, two key limitations persist. First, the literature has disproportionately emphasized instructional strategies and intervention effects, with limited attention given to how learner characteristics systematically influence CT through specific developmental pathways—such as the accumulation of digital competencies, the cross-domain transfer of cognitive skills (e.g., from mathematics to programming), and increased engagement in metacognitive strategies during technology-integrated tasks. Second, linear statistical models often fail to capture the complex, nonlinear interactions among the influencing factors (Ma et al. 2023). In summary, prior research has established that ICT use and learner background variables are significant predictors of CT. However, existing findings suggest that CT development is shaped by complex, context-dependent mechanisms that cannot be fully captured by linear statistical models (Aulia et al. 2025). This highlights the need for approaches capable of disentangling nonlinear and interactive effects among predictors. In line with prior evidence (Tamim et al. 2011), this study focuses on student individual characteristics and ICT usage contexts, which have been consistently identified as the most influential predictors of CT development. This emphasis is also directly aligned with the ICILS 2023 framework, which situates CT within a multi-layered structure of individual background and ICT usage (Fraillon and Rožman 2025). Thus, our focus is both theoretically guided and practically meaningful.

### 2.3. Applications of Educational Data Mining in CT Research

Data mining is a methodological approach for extracting valuable insights from large-scale datasets that holds significant promise in the field of educational research. Educational data mining refers to the use of data analytics techniques to explore and understand the vast amount of data generated during teaching and learning processes. It aims to uncover patterns in student learning behavior and instructional effectiveness to improve educational quality and enhance learning outcomes (Jiang et al. 2023; Lai 2022). By analyzing students’ learning data, educational data mining supports personalized learning, predicts academic performance, and provides real-time feedback, thereby enabling educators to make evidence-based decisions and implement targeted interventions that promote equity and optimize resource allocation (Chen et al. 2000). With the digitization of educational processes, data mining has become a key enabler of data-driven, precision-oriented, and personalized instruction, ultimately contributing to improved teaching efficiency and student achievement (Lee and Jang 2019). As the availability and complexity of educational data continue to grow, researchers increasingly apply data mining techniques to analyze student behavior, forecast academic success, and inform instructional strategies (Romero and Ventura 2020). One primary application of educational data mining is the prediction of student academic performance. Techniques such as decision trees, support vector machines, and neural networks have been employed to build predictive models based on variables such as academic scores, class participation, and homework completion to identify at-risk students and provide tailored interventions (Batool et al. 2023). Additionally, data mining facilitates personalized learning recommendations (Sarra et al. 2019). Methods such as collaborative filtering and association rule mining have been used to analyze students’ learning paths and to automatically suggest suitable learning resources, thus improving learning efficiency (Tatineni 2020). The integration of AI—particularly machine learning—and educational data mining is opening new opportunities for advancing educational research and innovation (Elahi et al. 2016; García et al. 2009).

In the domain of CT research, traditional studies have largely relied on classic statistical methods such as linear regression, SEM, and cluster analysis to examine the overall relationships among variables (Hsu et al. 2018). However, these methods are often limited in their ability to model high-dimensional data, nonlinear interactions, and complex interdependencies. As educational datasets become increasingly voluminous and complex, machine learning methods have emerged as powerful alternatives that offer superior modeling capacity and predictive performance, particularly in identifying nonlinear structures among the influencing factors. In recent years, the application of machine learning in educational data mining has increased. Researchers have adopted ensemble learning algorithms such as random forest to automatically identify the most predictive features and enhance model accuracy. For example, drawing on data from the ICILS 2018, one study employed supervised machine learning methods that used five classification algorithms to assess the predictive value of 22 school-level variables and ICT usage on student CT (Ezeamuzie et al. 2024). The findings indicated that although school-level ICT capabilities were not significant predictors, students from more affluent schools tended to score higher on CT assessments (Ezeamuzie 2024). Despite their predictive advantages, machine learning algorithms such as random forest and neural networks are frequently criticized as “black-box” models because of their lack of transparency and interpretability. This issue is particularly salient in educational research and practice, where actionable interventions require a clear understanding of how input variables contribute to learning outcomes.

To address this challenge, XAI techniques have been introduced into educational data mining. Among them, SHAP, a model-agnostic interpretability method grounded in cooperative game theory, has gained substantial recognition. SHAP quantifies the marginal contribution of each input feature to model predictions and visualizes these contributions, thereby enhancing the transparency of complex models (Lundberg and Lee 2017). Recent research has increasingly adopted SHAP-based methods to interpret machine learning models in education, particularly in STEM learning prediction (Alvarez-Garcia et al. 2024) and collaborative role recognition based on social, behavioral, and cognitive-emotional features (Wang and Xiao 2025). These studies demonstrate the potential of SHAP to enhance interpretability in educational contexts, but they also reveal certain limitations. Prior applications have largely been descriptive and context-specific, with limited integration into broader theoretical frameworks or policy-relevant domains. In particular, relatively little attention has been paid to contextualized predictors such as ICT usage across different learning settings, which are critical for understanding CT development.

Building on this background, the present study advances prior SHAP applications in two important ways. First, by leveraging the large-scale and internationally representative ICILS 2023 dataset, it moves beyond small-scale or localized studies, thereby enhancing both generalizability and policy relevance. Second, it expands SHAP’s role from post hoc interpretability to more explanation-oriented analysis, generating insights that strengthen both methodological advancement and educational relevance. In doing so, our study illustrates that integrating SHAP into large-scale educational data analysis can shift the focus from purely predictive modeling toward explanatory inquiry, thereby deepening the understanding of individual differences and their underlying mechanisms. This framing also directly informs our research questions, particularly regarding the nonlinear and context-dependent effects of ICT usage and student characteristics on CT development.

In summary, despite the growing adoption of machine learning and XAI in education, few studies have systematically examined how student characteristics and ICT usage interact in shaping CT. Addressing this gap, the present study employs a predictive and explanation-oriented framework to identify and interpret the most influential predictors of CT in an internationally representative dataset.

## 3. Methods

### 3.1. Data

The data for this study were drawn from ICILS 2023, which surveyed a total of 132,998 Grade 8 students from 35 countries and regions. The ICILS is designed to assess students’ readiness for life in a technology- and information-driven society. However, not all countries or students participated in the CT assessment. This study focused specifically on the subsample for which complete CT assessment data were available, comprising 81,871 students from 23 countries and one regional education system (North Rhine-Westphalia, Germany) (ICILS 2023). The CT assessment in ICILS 2023 was developed to evaluate students’ ability to transform real-world problems into computationally solvable forms, emphasizing skills in algorithm design and problem solving. The assessment consisted of four modules, each lasting 25 min, targeting two core dimensions of CT: problem conceptualization and solution operationalization.

- The problem conceptualization modules assessed students’ ability to understand and plan computational solutions using tools such as flowcharts, decision trees, and visual data representations. Students were asked to interpret scenarios and model systems in visual formats.
- The solution operationalization modules focused on students’ capacity to implement and execute computational solutions in a block-based programming environment. Students wrote and tested the code using an interactive visual interface that displayed execution outcomes.

Students’ CT performance was measured as the average of five plausible values, each representing a latent dimension of CT: abstraction, algorithmic thinking, automation, decomposition and debugging. The composite CT score was calculated as the mean of these five plausible values. According to the ICILS 2023 technical report, these scores were classified into five proficiency levels to reflect students’ developmental stages in CT. Level 1 represents 10% of students (*n* = 8187) who scored below 338.62. These students typically demonstrate minimal CT and can complete only the most basic tasks. Level 1 accounts for 24% of students (*n* = 19,649), with scores ranging from 338.62 to 446.67. Students at this level show limited but emerging CT and can understand basic algorithmic concepts. Level 2 is the largest group, comprising 37% of students (*n* = 30,292) who scored between 446.67 and 552.32. These students can solve common problems and apply moderately complex CT. Level 3 represents 23% of students (*n* = 18,830) who scored between 552.32 and 657.29. These students display strong CT and can design and implement efficient algorithmic solutions to complex problems. Level 4 is the highest proficiency level, comprising 6% of students (*n* = 4912), with scores above 657.29. Students at this level demonstrate exceptional CT and can develop innovative and optimized solutions for highly complex challenges.

In addition to CT scores, the ICILS student questionnaire collected extensive background information on students, including demographic characteristics and ICT usage. A total of 125 variables from the student questionnaire were used as potential predictors in this study, and students’ CT composite scores served as the outcome variable.

### 3.2. Statistical and Machine Learning Techniques

This section outlines the key statistical and machine learning techniques employed in this study.

#### 3.2.1. LightGBM

Light Gradient Boosting Machine (LightGBM) is an efficient gradient boosting framework based on decision tree algorithms. It enhances model performance by constructing multiple weak learners (decision trees) and optimizing them through a gradient boosting framework that minimizes a specified loss function. Compared with traditional gradient boosting decision tree methods, LightGBM incorporates several optimization strategies, such as histogram-based decision tree learning and leaf-wise tree growth, which significantly improve training speed and efficiency, especially when handling large-scale datasets with high-dimensional features (Ke et al. 2017). In this study, LightGBM was utilized to analyze feature importance to identify the key factors that predict the development of CT. Additionally, the SHAP method was included to provide interpretable model results.

#### 3.2.2. Random Forest

Random forest is an ensemble learning method introduced by Leo Breiman. It constructs a multitude of decision trees during training and outputs the mode of the classes (classification) or mean prediction (regression) of the individual trees. This approach enhances model stability and accuracy by reducing variance through the aggregation of multiple trees trained on different subsets of the data and features (Breiman 2001). In this research, random forest was applied to predict student CT performance. Feature importance analysis within the random forest framework helps determine the critical factors that contribute to the development of CT.

#### 3.2.3. SHapley Additive exPlanations

SHapley Additive exPlanations (SHAP) is a model-agnostic interpretability method proposed by Lundberg and Lee (2017). It is grounded in cooperative game theory and is utilized to quantify the contribution of each feature to the model’s predictions. SHAP provides a unified measure of feature importance and ensures consistency and local accuracy in explanations across various complex models, including LightGBM and random forest (Lundberg and Lee 2017). In the context of CT research, SHAP values facilitate the identification of key predictive factors at both the global and individual levels, enhancing the transparency and interpretability of data-driven educational studies.

### 3.3. Data Analysis

**Figure 1:**
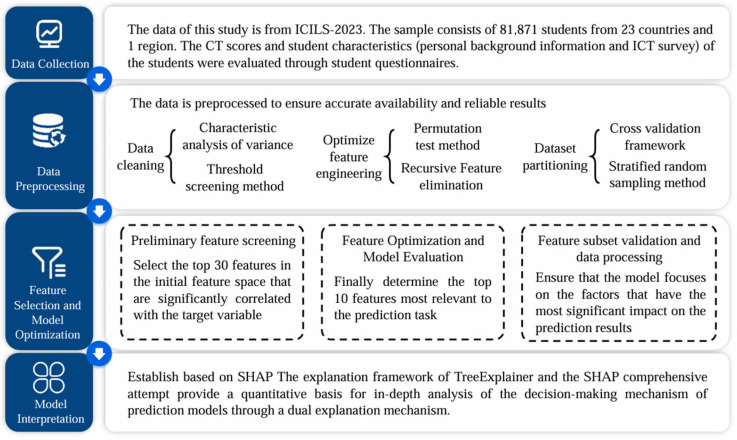
Research Procedure.

Following the principles of the educational data mining approach, this study adopted a systematic, multiphase data analysis process aimed at uncovering the meaningful relationships among student attributes and CT. Data mining is an iterative, empirically driven methodology that leverages logical and statistical procedures to discover patterns, predict outcomes, and support educational decision-making. The analysis in this study was structured into four main phases, as illustrated in Figure 1. The first phase, data acquisition, is detailed in Section 3.1. This methodological design is further supported by recent high-quality research, which has adopted comparable feature selection, independent model validation, and SHAP-based interpretation strategies (Zhang et al. 2025), thereby reinforcing the reliability of our approach.

#### 3.3.1. Data Preprocessing

To ensure data quality and analytical validity, rigorous preprocessing procedures were conducted. A threshold-based filtering method was first applied to address missing data: variables with more than 40% missing values were removed from the dataset to minimize systematic bias. To detect and handle anomalies, multidimensional outlier detection procedures, including analysis of variance, were implemented to eliminate features with zero variance. This ensured that the remaining data satisfied the statistical assumptions of normality and distributional consistency.

#### 3.3.2. Feature Selection and Model Optimization

A hierarchical and multistage feature engineering strategy was employed to enhance model performance and interpretability. Initial feature filtering was conducted using the feature importance scores generated by the LightGBM model. To mitigate the “curse of dimensionality” in high-dimensional spaces, we adopted permutation importance and recursive feature elimination to identify and remove low-impact variables. This process retained the top 30 predictive features (*p* < 0.05) that demonstrated significant correlations with CT outcomes, effectively reducing data redundancy.

A refined subset of 10 core features was subsequently determined using LightGBM in conjunction with GridSearchCV. Model performance was evaluated using the receiver operating characteristic (ROC) curve, which yielded a maximum area under the curve (AUC) of 0.72 on the test set. To validate both the stability and interpretability of the feature subset, SHAP values were computed to quantify the causal contributions of each feature at both the global and local levels.

Data splitting was conducted with stratified random sampling to preserve the class distribution, where 80% of the samples were assigned to the training set, and 20% were assigned to the test set. K-fold cross-validation was applied throughout to control the bias–variance trade-off. To further enhance model robustness, the synthetic minority oversampling technique was employed to address class imbalance, and Z-score normalization was applied to stabilize the variable distributions.

The feature selection process integrated filter, wrapper, and embedded methods to construct a five-category classification model with high predictive performance and strong generalizability across datasets.

#### 3.3.3. Model Interpretation

For the final explanatory analysis, a random forest classifier was optimized using GridSearchCV. The hyperparameters were tuned with the entropy criterion for node splitting, a maximum tree depth of 10, a minimum leaf sample size of 5, and an ensemble of 200 trees. A fixed random seed (random_state = 42) ensured experimental reproducibility. This configuration effectively balanced model complexity and generalization capability to yield enhanced predictive accuracy.

To interpret the model decisions, we implemented a SHAP-based explanation framework using shap. TreeExplainer. The SHAP values decompose the model output into additive feature contributions, where positive values indicate the features that increase the predicted CT score, and negative values indicate the suppressive effects. We utilized SHAP summary plots to visualize the influence of global features and ranked feature importance using the average absolute SHAP values (|SHAP|). This dual-level interpretive structure enabled both marginal effect estimation and interaction effect modeling across dimensions. By integrating SHAP’s cooperative game-theoretic explanations with ensemble modeling techniques, this study developed an end-to-end interpretable analytics pipeline—from feature engineering to decision explanation. This approach provides actionable insights for optimizing educational interventions and advancing evidence-based personalized learning.

At the methodological level, incorporating model interpretability into educational data mining practices offers a compelling paradigm for advancing XAI in education research.

The SHAP value for feature$\;X_{i\;}$is calculated as

$$\phi_{i}=\sum_{S\subseteq F\backslash \{i\}}\frac{\left|S\right|!\left(\left|F\right|-\left|S\right|-1\right)!}{\left|F\right|!}\left[f\left(S\cup \{i\}\right)-f\left(S\right)\right]$$

where

- *S* is a subset of the full feature set *F*, excluding feature *X_i_*;
- *f*(*S*∪{*i*}) is the model prediction when feature *X_i_* is included;
- *f*(*S*) is the model prediction without feature *X_i_*;
- The formula calculates the average marginal contribution of feature *X_i_* across all possible feature combinations.

## 4. Results

### 4.1. RQ1: Which Background Characteristics and ICT Usage Most Strongly Predict Students’ Computational Thinking?

**Figure 2:**
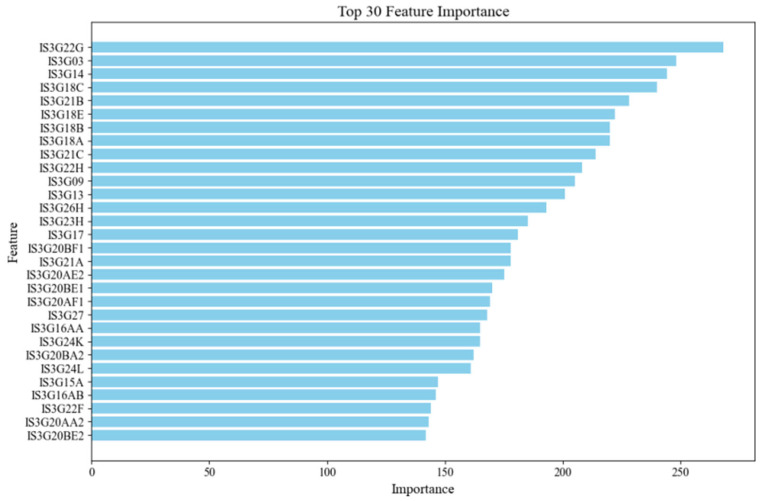
Top 30 Feature Importance Scores Identified by LightGBM.

To identify the most influential predictors of student CT performance, we first conducted feature ranking using the LightGBM model. Based on the model’s built-in feature importance scoring mechanism, the top 30 features most strongly associated with CT performance were selected. Figure 2 shows the importance distribution of these 30 features, with the length of each blue bar representing the relative contribution of each feature to the model’s predictive output.

**Figure 3:**
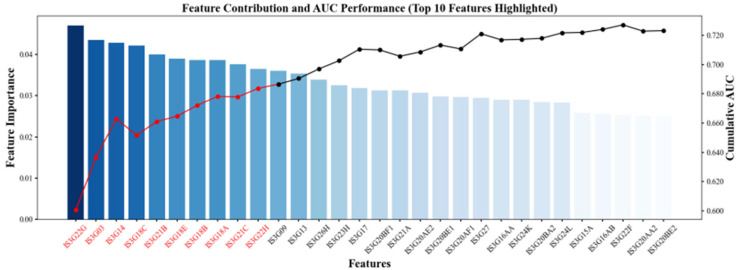
SHAP and AUC-Based Evaluation of the Top 10 Key Predictive Features.

Building upon the initial selection, we further validated and refined the feature set using the random forest model with cross-validation and a performance assessment. By comparing the number of features retained with the corresponding changes in the AUC, we identified the minimal feature subset that maintained a satisfactory model performance. Consistent with recent evidence (Tan et al. 2024), which emphasized that including too many features can undermine model interpretability and highlighted the importance of balancing “parsimony” and “explanatory power,” we retained the final 10 key features. Figure 3 presents the final 10 key features, along with their relative importance, in the prediction task. The left panel displays the mean SHAP values for each feature and indicates the marginal contribution of this variable to the model’s output. The right panel shows the AUC drop resulting from removing each feature, with red markers denoting the top 10 most impactful predictors.

**Figure 4:**
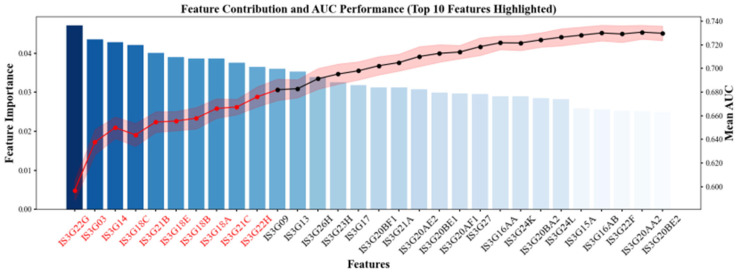
Top 10 Feature Contributions to AUC across a 10-Fold Cross-Validation.

To assess the robustness of the feature subset, we conducted a 10-fold cross-validation analysis. As shown in Figure 4, the red dots represent the top 10 features that contributed most significantly to the AUC gains across the different folds. The consistent performance of these features across data splits demonstrates the predictive stability and generalizability of the selected variable set.

Ultimately, the final 10 key features exhibited high predictive power across multiple models and validation datasets. Detailed descriptions of these variables, including their categories and definitions, are provided in Appendix A.

### 4.2. RQ2: How Do the Most Influential Features Affect Students’ Computational Thinking?

**Figure 5:**
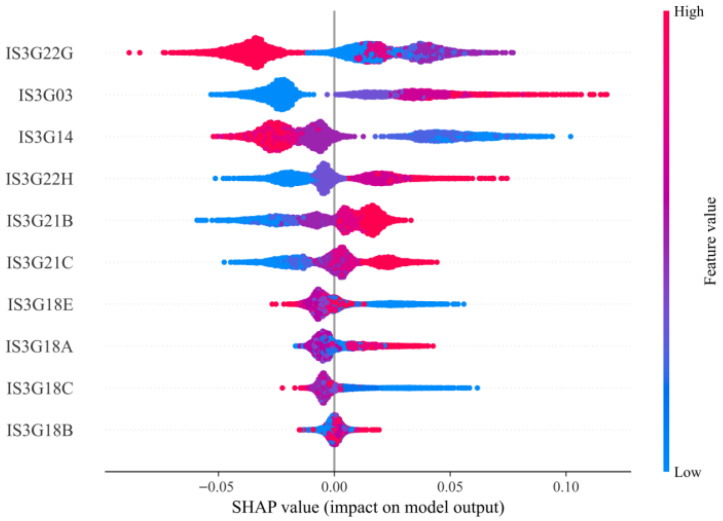
SHAP Summary Plot: Average Marginal Contributions of the Key Features of CT Prediction.

To gain deeper insights into how the key features predict student CT performance, we applied SHAP for model interpretability analysis. Figure 5 presents the SHAP summary plot, which ranks the top 10 most impactful features based on their average marginal contributions across all samples. The Y-axis lists the final set of the top-ranked features identified by the model: IS3G22G, IS3G03, IS3G14, IS3G22H, IS3G21B, IS3G21C, IS3G18E, IS3G18A, IS3G18C, and IS3G18B. The X-axis represents the SHAP value of each feature, ranging from approximately −0.10 to +0.10, and indicates the direction and magnitude of each feature’s contribution to the model’s prediction.

Each dot in the plot represents a SHAP value for a given feature in a specific student sample. The color gradients denote the original feature values: blue represents lower values, and red represents higher values. The horizontal position of each dot corresponds to its SHAP value and specifies whether this feature increased or decreased the predicted CT score. The violin-shaped spread reflects the distribution and density of the SHAP values for each feature across the dataset. The results from Figure 5 demonstrate that the predictive contributions of individual features on CT performance are nonlinear. For example, IS3G22G—which reflects students’ frequency of ICT usage in information technology courses—exhibits a distinctly nonlinear pattern. The red dots (indicating higher values) are concentrated toward the left-hand side of the X-axis and peak near negative SHAP values. This finding indicates that frequent ICT usage in class may have a negative effect on predicted CT, suggesting a potential diminishing return or even a negative association. In contrast, the blue dots (representing lower ICT usage) are mostly centered on zero, which shows that infrequent ICT use has a weak or neutral effect on model predictions.

**Figure 6:**
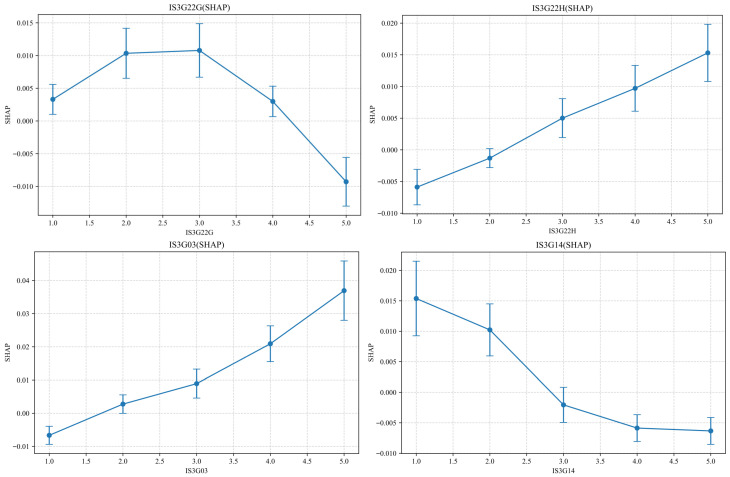

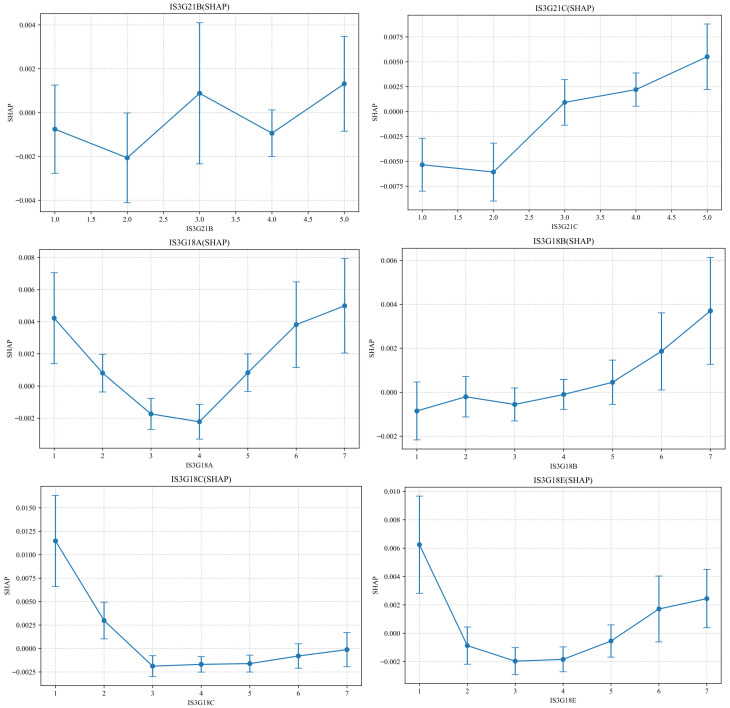
SHAP Dependence Plots: Feature-Specific Effects on Model Prediction.

To further examine these nonlinear effects, SHAP dependence plots were generated for each of the 10 key features (Figure 6) to illustrate the relationships between the feature values and their SHAP contributions and to highlight potential thresholds and nonlinear trends.

In each plot, the X-axis represents the raw feature values, and the Y-axis identifies the corresponding SHAP values. Several features demonstrate clear nonlinear trajectories, including U-shaped, inverted U-shaped, or monotonic relationships. These patterns suggest that, in educational contexts, simply increasing the frequency of certain types of ICT usage does not guarantee improved CT performance. Rather, it is crucial to identify the optimal ranges or thresholds of ICT engagement where positive effects are maximized and negative effects are avoided.

To further align the SHAP visualizations with the semantic meanings of the original questionnaire items, we conducted a detailed interpretation of the SHAP trends for each of the 10 key predictive features. These insights reveal differentiated and sometimes nonlinear mechanisms by which student characteristics and ICT usage contribute to the CT performance predictions.

IS3G22G and IS3G22H: IS3G22G refers to the frequency of ICT use in computer-related school courses, such as Information technology, computer studies or similar subjects taught in the school curriculum. The SHAP dependence plot displays an inverted U-shaped trend, with moderate ICT usage frequencies (“In some lessons”; values 3) corresponding to the highest SHAP values. This suggests that moderate engagement with ICT in these courses yields the most positive predictive contribution, while extremely low or very high usage results in reduced contributions. IS3G22H reflects the frequency of ICT use in applied or vocational school courses. The SHAP values show a monotonically increasing trend, indicating that higher usage frequencies (“In every or almost every lesson”; values of 5) consistently enhance CT performance predictions, which highlights the benefits of practical, application-oriented ICT usage. However, importantly, IS3G22H, as defined in the ICILS 2023 questionnaire, does not differentiate between specific vocational domains (e.g., informatics, health care, or applied arts). Therefore, the interpretation of this trend should be approached with caution, as the potential variability in ICT integration across different vocational fields should be recognized.

IS3G03: This variable reflects students’ educational aspirations. The SHAP values increase steadily with higher aspiration levels and demonstrate a positive linear relationship. Students with higher future academic expectations are more likely to exhibit stronger predicted CT performance. Although this result may reflect greater motivation or self-regulation, it may also be related to unmeasured factors such as cognitive ability. This interpretation should therefore be treated with caution.

IS3G14: IS3G14 measures the number of books available at home. Surprisingly, this feature shows a negatively sloped SHAP trend, signifying that students from households with more books contribute less to the prediction of CT performance. This counterintuitive pattern may suggest a divergence between traditional literacy environments and digitally mediated CT development.

IS3G21B and IS3G21C: IS3G21B denotes the frequency with which students actively post or view content on social media platforms (e.g., Instagram, TikTok, and Snapchat) while completing academic tasks outside of school. The SHAP values for this variable fluctuate and show no clear linear pattern, although a slightly increasing tendency is observed. This indicates unstable and context-dependent effects on the CT predictions. IS3G21C, in contrast, refers to passive behaviors such as viewing updates or checking feedback on their posts. This factor shows a clear positive trend and suggests that frequent passive engagement with social media while studying is associated with higher CT prediction values. This may reflect enhanced digital multitasking or metacognitive monitoring abilities.

IS3G18A, IS3G18B, IS3G18C and IS3G18E: These four variables reflect ICT usage frequency under varying temporal (school day vs. non-school day), spatial (in-school vs. out-of-school), and functional (academic vs. non-academic) conditions. IS3G18A (academic use on school days, in school) and IS3G18E (academic use on non-school days, out of school) both exhibit U-shaped SHAP patterns. That is, very low and very high frequencies of usage contribute positively to CT predictions, whereas moderate frequencies yield relatively lower SHAP values. This may represent the presence of an optimality threshold or that both intense and minimal engagement foster CT development in different ways. IS3G18C (academic use on school days, out of school) presents a monotonically decreasing trend. As the usage frequency increases, the SHAP value decreases, indicating that frequent off-campus academic ICT usage may not effectively support CT development, potentially because of distractions or a lack of structured support. IS3G18B (non-academic ICT use on school days, at school) shows a monotonically increasing trend and implies that students’ frequent engagement in informal or recreational ICT activities within school settings positively contributes to their predicted CT performance. This finding hints both at the potential positive spillover effects of informal digital practices—such as exploration, creativity, or social interaction—on students’ strategic use of digital tools and at broader information literacy.

## 5. Discussion

In the context of increasingly data-rich educational environments, the integration of XAI into educational data mining offers powerful tools for revealing the mechanisms underlying students’ CT development. Compared with conventional approaches that rely primarily on linear regression or SEM—which are often inadequate for capturing the complexity of the interactions between predictive variables and CT—this study employs a hybrid framework that combines LightGBM and SHAP based on ICILS 2023 large-scale international assessment data. The model identifies the key factors that are associated with CT and reveals their nonlinear relationships, such as U-shaped, inverted U-shaped, and monotonic trends. This methodological approach improves both predictive performance and interpretability, offering a new pathway for understanding individual student differences and designing targeted educational interventions.

The findings indicate that the effect of ICT usage on CT performance depends heavily on the instructional context. In computer-related school courses (IS3G22G), the SHAP values follow an inverted U-shaped pattern, with moderate ICT usage associated with the most positive predictive contributions. This trend supports the “marginal effect of technological intervention” hypothesis (Selwyn 2021), which suggests that beyond a certain point, excessive ICT engagement—particularly in non-programming activities such as passive information consumption—may reduce opportunities for deep cognitive processing and hinder CT development (Sirakaya 2020). In contrast, applied or vocational courses (IS3G22H) show a monotonically increasing SHAP trend and indicate that more frequent ICT usage consistently contributes to CT performance. This may be because vocational courses often integrate ICT into hands-on, task-oriented learning—such as digital design, simulations, or technical applications—where greater engagement directly supports skill acquisition and CT-related problem solving (Grover and Pea 2013). The ICILS does not specify which vocational fields were involved, but this trend suggests that structured, purposeful ICT integration into authentic, practice-oriented contexts may be particularly effective. Additionally, evidence from related research confirms that participation in programming, robotics, or data analytics courses is positively associated with CT development (Li et al. 2023; Nouri et al. 2020; Weintrop et al. 2016), which reinforces the importance of pedagogical design over mere frequency of use. Together, these findings underscore the need for context-sensitive ICT implementation that aligns with cognitive demands and learning goals (Mills et al. 2024).

The analysis also reveals a significant positive association between students’ educational expectations (IS3G03) and their CT performance. The SHAP value analysis shows that students who aspire to attain higher levels of education tend to exhibit stronger CT abilities. This finding aligns with existing academic research, which suggests that higher educational aspirations motivate students to actively cultivate the cognitive and problem-solving skills necessary for future academic and career success (Abdrakhmanov et al. 2024). Educational expectations play a critical role in promoting both academic achievement and cognitive development. By fostering stronger intrinsic motivation and self-regulated learning behaviors, higher expectations encourage students to engage more deeply in the skill acquisition processes that are essential for CT development (Tariq et al. 2025). Furthermore, studies have shown that students with elevated educational goals tend to place greater emphasis on cultivating academic competencies, including CT skills, thus laying a strong foundation for future advancements in fields such as STEM (Siu-cheung and Gautam 2019).

A small but significant negative association is observed between the number of books at home (IS3G14) and students’ predicted CT performance. In educational research, this indicator is often used as a proxy for cultural capital and refers to the symbolic resources and literacy-based assets—such as access to books, parental education, and the intellectual environment—that can facilitate academic success (Bourdieu et al. 2011). However, this measure is broad and does not capture the types or functions of the books. It is plausible that if the home library is composed primarily of fiction, leisure reading, or non-technical content (e.g., novels and poetry), it may not directly support the cognitive demands of CT. In contrast, access to technical books, STEM-oriented materials, or problem-solving manuals could offer more relevant support for CT development. Therefore, the observed negative association should not be interpreted as suggesting that books are inherently counterproductive for CT but rather that traditional forms of cultural capital may not fully align with the digital and analytical skills emphasized in CT. This highlights the growing importance of digital capital—resources such as programming tools, digital platforms, and interactive content—in shaping students’ cognitive readiness for 21st-century problem-solving tasks (Cortoni 2025; Vrieler 2017). Future research would benefit from including qualitative indicators of the book type and function to provide more precise insights into how different forms of home literacy resources relate to CT skills.

A nuanced pattern emerges regarding students’ social media usage behaviors. Specifically, when students frequently check social media updates in general (IS3G21B), this positively contributes to the prediction of CT performance. Although counterintuitive at first glance, this outcome may reflect adaptive digital habits formed through the daily interaction with dynamic information environments (Wilmer et al. 2017). Prior research suggests that such strategic digital monitoring may foster skills in information triage, relevance evaluation, and cognitive flexibility (Livingstone and Helsper 2007), which are foundational to metacognition and CT (Flavell 1979). In contrast, when students frequently check social media while studying or working, this presents a negative association with CT performance. This behavior is more likely to reflect task-switching interference and divided attention, in line with passive attention interference theory (Junco 2012). Engaging with social media during cognitively demanding activities may increase cognitive load (Holmberg 2016) and disrupt sustained reasoning processes (Kirschner and De Bruyckere 2017), which are critical for CT. This apparent contradiction can be reconciled by distinguishing the intent and context of the behavior: checking updates during downtime may enhance students’ metacognitive efficiency, whereas checking updates during learning tasks likely fragments attention and impairs performance. These findings support a more differentiated view of digital behavior and emphasize that social media use is not inherently detrimental but is dependent on its timing, purpose, and regulation (Montag and Markett 2023). Educational interventions should focus not only on limiting media use but also on guiding students toward selective, self-regulated engagement that leverages digital habits to enhance CT development (Herro et al. 2021; Hsu et al. 2018; Junco 2012).

Students’ ICT usage across different temporal (school days vs. non-school days), spatial (in-school vs. out-of-school), and purpose-driven (academic vs. non-academic) contexts exhibits significant heterogeneity in their predictive contributions to CT performance.

First, academic ICT use in school on school days and outside school days both show a U-shaped SHAP trend. In these contexts, students with either very low or very high usage frequencies contribute more positively to CT predictions, whereas those with moderate usage frequencies present lower SHAP values. This pattern may suggest the presence of a subset of moderate-frequency users whose ICT engagement lacks specific learning goals, strategic regulation, or cognitive depth—thus possibly reflecting more passive or habitual usage (Liu et al. 2024). However, we acknowledge that passive or unproductive engagement can also occur among low- and high-frequency users and that usage frequency alone cannot reliably determine cognitive quality. The inference here is based on the SHAP value dip, not direct behavioral evidence. Therefore, this interpretation should be viewed as hypothesis-generating, which underscores the need for future research to integrate qualitative measures of user intent, task type, and learning strategies. Repetitive operations or shallow information retrieval, regardless of frequency, may yield limited cognitive benefits (Lim and Chai 2008).

Second, academic ICT use outside of school on school days exhibits a monotonically decreasing SHAP trend, where higher usage frequencies are linked to lower CT predictions. This pattern suggests that frequent academic ICT use in unsupervised or unstructured environments may suffer from insufficient instructional guidance, resource constraints, or poor task clarity (Palop et al. 2025), thereby reducing the efficacy of ICT as a CT support tool. This interpretation aligns with concerns about unstructured ICT use increasing cognitive load and disrupting deep processing (Kirschner and De Bruyckere 2017).

Third, non-academic ICT use at school on school days demonstrates a gradual positive SHAP trend, with higher usage frequencies corresponding to stronger CT predictions. Although these activities do not serve explicit academic purposes, they may indirectly support CT development by promoting exploratory learning, digital multitasking, and resource integration through informal peer interactions or self-directed engagement (Flavell 1979; Junco 2012). This suggests that not all non-academic ICT use is detrimental—when purposefully engaged, it can function as a latent pathway to develop digital cognitive literacy (Ferguson et al. 2015; Livingstone and Helsper 2007).

Accordingly, these findings reinforce the fact that the educational value of ICT usage depends not only on frequency but also, more critically, on contextual structures, student motivations, and the alignment of technology use with cognitive goals (Wang and Hannafin 2005). Educational interventions should emphasize goal-directed ICT engagement and embed technology use into cognitively rich tasks to ensure meaningful and context-sensitive support for CT development (Palop et al. 2025).

## 6. Conclusions

As CT gains importance as a core educational competency, understanding its influencing factors is essential. Using ICILS 2023 data, this study applies an explainable AI approach—combining LightGBM and SHAP—to identify the key predictors of CT performance and uncover their nonlinear effects. Ten influential variables are identified, including educational expectations, the home literacy environment, social media use, and ICT usage in different contexts. The findings reveal that the effect of ICT on CT varies according to the purpose and setting: moderate ICT use in computer-related courses yields the most positive effects, while excessive ICT use may reduce the cognitive benefits. In contrast, ICT use in vocational courses shows a consistently positive trend. Differences in social media behavior also emphasize the role of goal orientation and self-regulation. This study demonstrates the value of SHAP in improving model interpretability and identifying complex patterns missed by traditional methods. This research thus provides actionable insights for integrating ICT purposefully into instruction to support CT development. Future research should further explore the contextual mechanisms by using longitudinal and cross-cultural data.

## 7. Limitations

Although this study leverages large-scale international data and employs a hybrid approach that combines machine learning and XAI to systematically identify the key predictors of students’ CT and uncover complex nonlinear mechanisms, several limitations should be acknowledged. First, the ICT usage data are primarily derived from self-reported student questionnaires, which may be subject to social desirability bias. Future studies could enhance data accuracy and interpretability by incorporating objective behavioral data, such as usage duration, the platform type, or interaction logs. Second, although the dataset includes a large and diverse student population, the current findings may not fully capture the contextual nuances. There remains a need for stratified analyses across age groups, cultural contexts, and educational systems to validate the model’s generalizability and cross-contextual transferability. Third, the study employs a cross-sectional design, which limits causal inference. To better understand the dynamic interactions between ICT usage and CT development, longitudinal studies or experimental designs are warranted. Additionally, future research could expand the application of XAI techniques in educational assessment and learning trajectory modeling (Tiukhova et al. 2024) and incorporate multimodal behavioral data for fine-grained modeling to improve both the explanatory power and predictive accuracy (Chango et al. 2021).

## Data Availability

Restrictions apply to the availability of these data. Data were obtained from [ICILS 2023 database] and are available at [https://www.iea.nl/studies/iea/icils/2023 (accessed on 10 March 2025)] with the permission of [ICILS 2023 database].

## Abbreviations

The following abbreviations are used in this manuscript:

CT	Computational thinking
ICT	Information and Communication Technology
XAI	Explainable Artificial Intelligence

## Appendix A

### Final Set of the Top 10 Predictive Features

**Variable ID**	**Item Content**	**Source**
IS3G22G	At school, how often do you use ICT during lessons in the following subjects or subject areas? [Information technology, computer studies or similar]	ICT Questionnaire
IS3G03	What is the highest level of education you expect to complete?	Background Questionnaire
IS3G14	About how many books are there in your home?	Background Questionnaire
IS3G22H	At school, how often do you use ICT during lessons in the following subjects or subject areas? Practical or vocational [Add any appropriate national examples]	ICT Questionnaire
IS3G21B	Outside of school, how often do you do the following activities not related to your schoolwork at the same time as doing your schoolwork? Use social media (e.g., [Instagram, TikTok and Snapchat]) to post or view content	ICT Questionnaire
IS3G21C	Outside of school, how often do you do the following activities not related to your schoolwork at the same time as doing your schoolwork? Check social media for new posts or responses to my posts	ICT Questionnaire
IS3G18E	How often do you use ICT in these places? (On non-school days) Outside of school for schoolwork	ICT Questionnaire
IS3G18A	How often do you use ICT in these places? (On school days) At school for schoolwork	ICT Questionnaire
IS3G18C	How often do you use ICT in these places? (On school days) Outside of school for schoolwork	ICT Questionnaire
IS3G18B	How often do you use ICT in these places? (On school days) At school for other purposes	ICT Questionnaire
